# Topical povidone iodine inhibits bacterial growth in the oral cavity of patients on mechanical ventilation: a randomized controlled study

**DOI:** 10.1186/s12903-020-1043-7

**Published:** 2020-02-24

**Authors:** Shoma Tsuda, Sakiko Soutome, Saki Hayashida, Madoka Funahara, Souichi Yanamoto, Masahiro Umeda

**Affiliations:** 1 Department of Clinical Oral Oncology, Nagasaki University Graduate School of Biomedical Sciences, 1-7-1 Sakamoto, Nagasaki, 852-8588 Japan; 2 Oral Care Center, Nagasaki University Hospital, 1-7-1 Sakamoto, Nagasaki, 852-8588 Japan; 3 Kyushu Dental University School of Oral Health Sciences, 2-6-1 Manazuru, Kokurakitaku, Kitakyushu, 803-8580 Japan

**Keywords:** Mechanical ventilation, Oral bacteria, Oral care, Povidone iodine, Saliva

## Abstract

**Background:**

Topical 0.12% chlorhexidine has been used widely to prevent ventilator-associated pneumonia in patients undergoing mechanical ventilation. However, it is not approved for mucosal application in Japan. The aims of this study were to investigate if topical povidone iodine (i) inhibits bacterial growth and (ii) disrupts the balance of the oral microbiota.

**Methods:**

This randomized controlled clinical trial included 23 patients who underwent mechanical ventilation in the intensive care unit. The patients were divided randomly into two groups: the intervention group (*n* = 16) and the control group (*n* = 7). All patients received oral cleaning with 3% hydrogen peroxide, followed by irrigation with tap water. The patients in the intervention group received 10% povidone iodine applied topically to the oral cavity. The concentration of total bacteria in the oropharyngeal fluid were determined before, immediately after, 1 h, 2 h, and 3 h after oral care using the Rapid Oral Bacteria Quantification System, which is based on dielectrophoresis and impedance measurements. The number of streptococci, methicillin-resistant *Staphylococcus aureus*, *Streptococcus pneumoniae*, *Pseudomonas aeruginosa*, *Porphyromonas gingivalis*, and *Candida albicans* before, immediately after, 1 h, and 3 h after oral care were estimated based on real-time polymerase chain reaction data.

**Results:**

After irrigation of the oral cavity, the number of bacteria decreased, but increased again at 1 h after oral care in the control group; however, in the intervention group, the concentration of bacteria was significantly lower than that in the control group at 1 hour (*p* = 0.009), 2 h (*p* = 0.001), and 3 h (*p* = 0.001) after oral care. The growth of all bacterial species tested was inhibited in the intervention group at 3 h after oral care, suggesting that povidone iodine did not disturb the balance of the oral microbiota.

**Conclusions:**

Topical application of povidone iodine after cleaning and irrigation of the oral cavity inhibited bacterial growth in the oropharyngeal fluid of patients on mechanical ventilation while not disrupting the balance of the oral microbiota.

**Trial registration:**

University Hospitals Medical Information Network Clinical Trials Registry (UMIN-CTR), UMIN000028307. Registered 1 September 2017.

## Background

Ventilator-associated pneumonia (VAP) is an airway infection developing more than 48 h after intubation that affects 8–28% of patients requiring mechanical ventilation. VAP is a major complication in the intensive care unit that has been reported to contribute to higher mortality rates and longer hospital stays [1–4].

There are several risk factors for VAP, and some prevention strategies have been explored. One of the main causes of VAP is thought to be the aspiration of oral bacteria. The US Institute for Healthcare Improvement (IHI) recommends a preventive intervention called the IHI Ventilator Bundle, which consists of 1) elevation of the head of the bed, 2) daily sedation vacations and assessment of readiness to extubate, 3) peptic ulcer disease prophylaxis, 4) deep vein thrombosis prophylaxis, and 5) daily oral care with 0.12% chlorhexidine [5]. However, despite its effectiveness in preventing VAP, 0.12% chlorhexidine is not approved for mucosal application in Japan. Therefore, oral care is not included in the Japanese Society of Intensive Care Medicine (JSICM) VAP Bundle [6].

As an alternative to 0.12% chlorhexidine, povidone iodine has been used widely to disinfect the mucous membranes in Japan. However, it is not generally used to prevent VAP. According to the meta-analysis by Labeau et al. [7] on prevention of VAP by oral antiseptics, a significant reduction was observed in patients receiving chlorhexidine, while in those receiving povidone iodine the efficacy on preventing VAP was unclear because of fewer number of studies. The aims of this randomized controlled study were to investigate if topical povidone iodine (i) inhibits bacterial growth and (ii) disrupts the balance of the oral microbiota in the oral cavity of patients undergoing mechanical ventilation.

## Methods

### Patients

This is a randomized phase II trial conducted before a large-scaled phase III study with the onset of that VAP as the primary endpoint. This study adheres to CONSORT guidelines. The primary endpoint of the current study is the difference in the number of total bacteria in the oropharyngeal fluid between patients receiving standard oral care and those treated with topical povidone iodine in addition to oral care at 3 h after intervention. From the results of our previous study, assuming that logarithm of number of bacteria in the oropharyngeal fluid after 3 h in the control group is 7.0 ± 0.8 cfu/mL and it reduces to 6.0 cfu/mL in the intervention group, when assigned by 2:1, alpha error is 0.05, and power is 80%, the required number of cases is 24 cases. The allocation will be determined by data manager responsible for biostatistical analysis. This open-labeled, randomized controlled study included 23 patients who received mechanical ventilation in the intensive care unit of Nagasaki University Hospital between April and September 2018. Inclusion criteria are 20–90 years old patients with ventilator by oral intubation. One patient who could not collect saliva due to dry mouth was excluded from the study. Informed consent to participate was obtained in writing for patients admitted for surgery, but in some patients for emergency admission verbally from family members.

### Consent for publication

#### Intervention

**Fig. 1:**
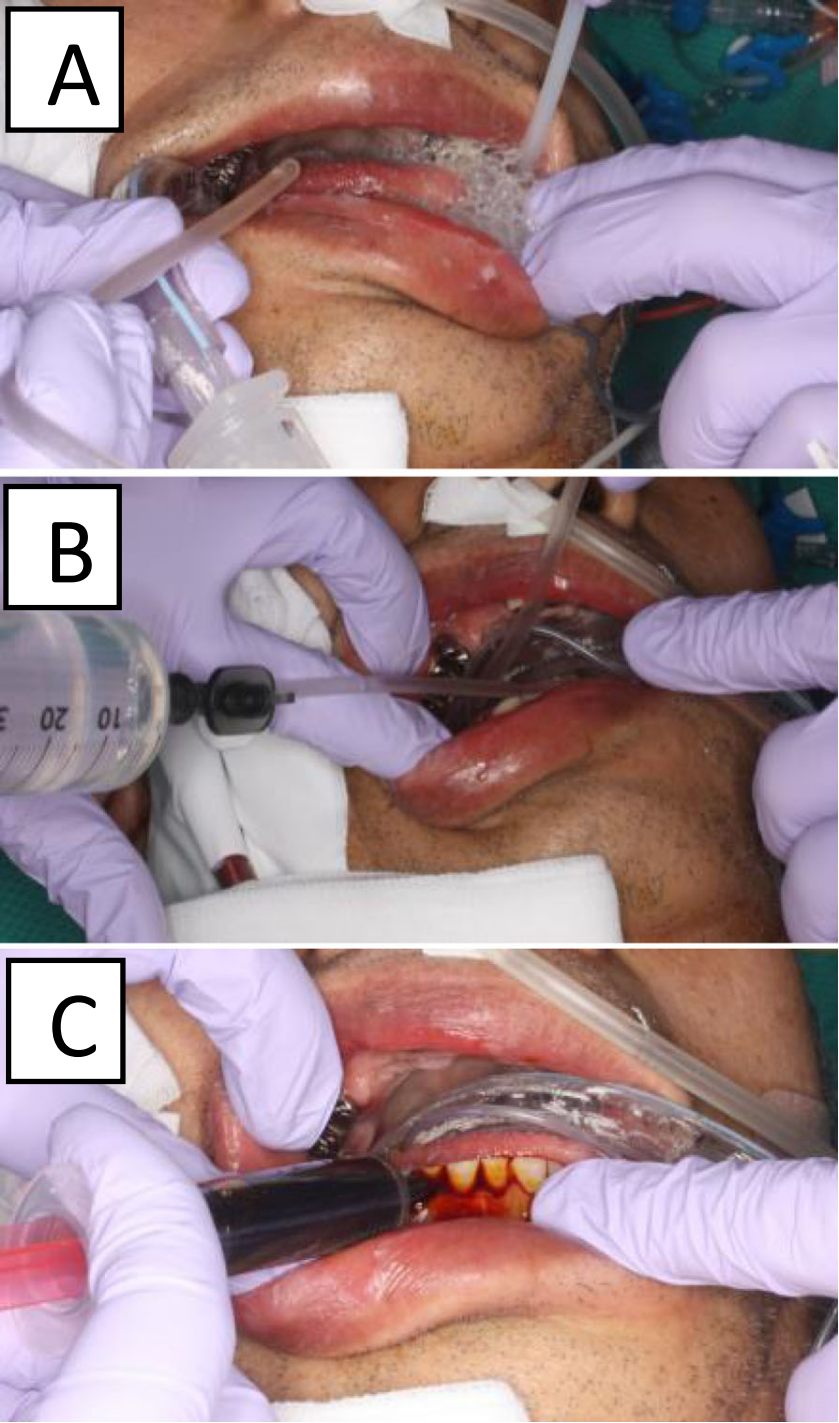
Oral care method. **a** Wiping of the oral mucosa with 3% hydrogen peroxide; **b** irrigation with tap water; **c** topical application of 10% povidone iodine

All patients received oral care by a dentist and dental hygienist at the same time every day. Oral care consisted of wiping with 3% hydrogen peroxide (Oxydol; KENEI Pharmaceutical Co.,Ltd., Osaka, Japan) and irrigation with 200 mL of tap water plus suction. The patients in the intervention group received 5 mL of 10% povidone iodine (Isodine; Shionogi Seiyaku Co.,Ltd., Tokyo, Japan) applied topically in the oral cavity (Fig. 1). 5 ml of povidone iodine was dripped into the oral cavity including the gingiva, tongue and buccal mucosa using a syringe. Care was taken with suction through the oropharynx to prevent the patient from aspirating the tap water or povidone iodine. After washing, suction was performed through the side tube of the tracheal cannula.

#### Measurement of the concentration of total bacteria in the oropharyngeal fluid by the rapid Oral Bacteria quantification system

The concentration of total bacteria in the oropharyngeal fluid was determined using the Rapid Oral Bacteria Quantification System (Panasonic Healthcare Co. Ltd., Osaka, Japan), which is based on dielectrophoresis and impedance measurements [8, 9]. To collect the samples, a cotton swab was immersed in oropharyngeal fluid for 5 s before oral care and 1 min, 1 h, 2 h, and 3 h after oral care. Next, to determine the bacterial count, the cotton swab was inserted into the apparatus.

#### Estimation of the number of some oral microorganisms by real-time polymerase chain reaction (PCR)

To determine changes in the balance of the oral microbiota in the intervention group, 0.1–0.2 mL of oropharyngeal fluid were collected with a syringe before oral care and 1 min, 1 h, and 3 h after oral care. Genomic DNA from oropharyngeal fluid was isolated using a DNA extraction kit (InstaGene Matrix; Bio-Rad Laboratories, Hercules, CA, USA) according to the manufacturer’s instructions. After adding 200 μL of InstaGene Matrix to the precipitate and incubating at 56 °C for 30 min, the sample was stirred and incubated at 100 °C for 8 min. All samples were stored at − 20 °C after the above processing. Samples were thawed immediately prior to quantitative real-time PCR and centrifuged at 10,000×*g* for 10 min at 4 °C. The supernatant was used to estimate the number of bacteria and generate the standard calibration curve for quantitative real-time PCR.

**Fig. 2:**
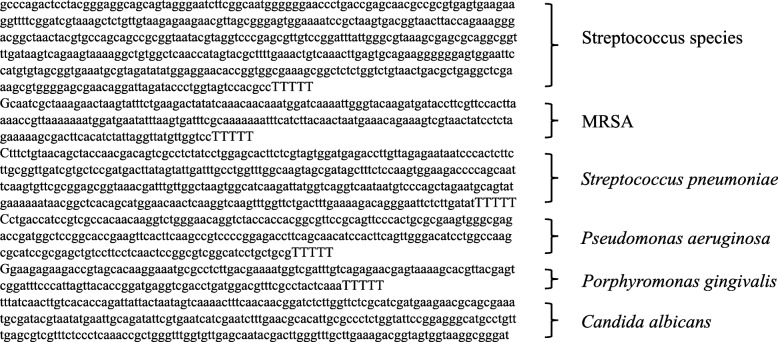
Artificial DNA sequences used in real-time polymerase chain reaction

The concentration of streptococci, methicillin-resistant *Staphylococcus aureus* (MRSA), *Streptococcus pneumoniae*, *Pseudomonas aeruginosa*, *Porphyromonas gingivalis*, and *Candida albicans* were estimated based on real-time PCR data. For the standard calibration curve for quantitative real-time PCR, the DNA sequence of the target microorganism was synthesized, and artificial DNA was used (Fig. 2). The reaction solution (total volume, 20 μL) contained 10 μL of KOD SYBR® qPCR Mix (TOYOBO Co.,Ltd., Osaka, Japan), 1 μL of oropharyngeal fluid DNA sample, 3 μL of primers for each target microorganism (Table 1), and 6 μL of deionized water per well. After the initial heat denaturation at 98 °C for 2 min, the target DNA was amplified by carrying out 40 cycles of two steps: 95 °C for 20 s (heat denaturation) and 62 °C for 90 s (annealing). After completion of amplification, fluorescence signals were detected at 95 °C for 15 s, 60 °C for 30 s, and 95 °C for 15 s to generate a melting curve, and the specificity of the amplified product was confirmed. Data were analyzed using Thermal Cycler Dice® Real-time System software (TaKaRa BIO Inc., Shiga, Japan). The concentration of microorganisms in oropharyngeal fluid was the copy number estimated based on the amplification and calibration curves.

**Table 1:** Primer used in the study

Terget	Gene	Sequence	Size
Total *Streptococci*	16S rRNA	TCGGATCGTAAAGCTCTGTTGTA GGACAACGCTCGGGACCTAC	137
MRSA	MecA	GCAATCGCTAAAGAACTAAG GGGACCAACATAACCTAATA	222
*Streptococcus pneumoniae*	ply	ATTTCTGTAACAGCTACCAACGA GAATTCCCTGTCTTTTCAAAGTC	348
*Pseudomonas aeruginosa*	gyrB	CCTGACCATCCGTCGCCACAAC CGCAGCAGGATGCCGACGCC	222
*Porphyromonas gingivalis*	rpoB	GGAAGAGAAGACCGTAGCACAAGGA GAGTAGGCGAAACGTCCATCAGGTC	143
*Candida albicans*	ITS1	TTTATCAACTTGTCACACCAGA ATCCCGCCTTACCACTACCG	273

*Abbreviations*: *MRSA* methicillin-resistant *Staphylococcus aureus*

### Statistical analysis

The data of patients characteristics were analyzed by means of descriptive statistics and inferential statistics. The differences between total bacterial counts in the intervention and control groups were analyzed by the Mann–Whitney U-test, using SPSS software (version 24.0; Japan IBM Co., Ltd., Tokyo, Japan).

## Results

### Patient characteristics

The patient characteristics were shown in Table 2.

**Table 2:** Patient characteristics

Factor	Category	Intervention group	Control group
Sex	male	10	4
	female	5	4
Age	mean	63.5 years	65.8 years
Reason of ventilation	sepsis shock	5	4
	surgery	2	3
	pneumonia	3	0
	heart disease	2	0
	stroke	1	0
	liver disfailure	1	0
	cardiopulmonary arrest	0	1
	multiorgan disfailure	1	0
Duration of ventilation	mean	6.1 days	5.5 days

### Total bacterial count in the oral cavity of the intervention and control groups

**Fig. 3:**
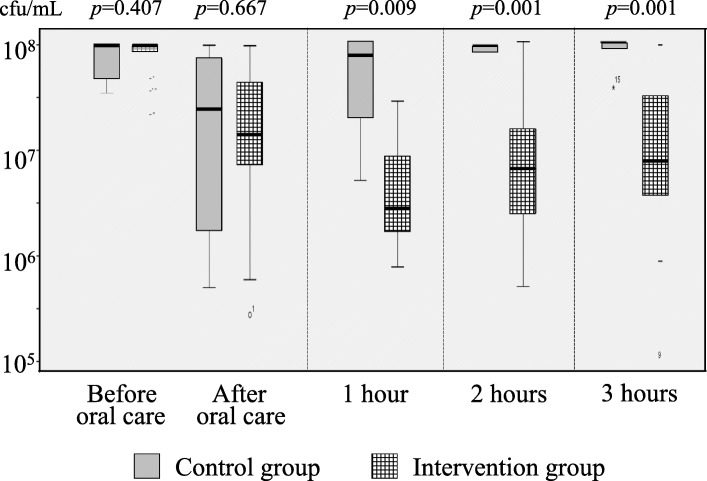
Changes in the total bacterial count before and after oral care in the intervention and control groups. There was no significant difference in the total bacterial count between the 2 groups before and after oral care, but at 1, 2, and 3 h after application of iodine povidone, it in the intervention group was significantly lower than that in the control group

Figure 3 shows the changes in the total bacterial count before and after oral care in the intervention and control groups. After irrigation of the oral cavity, the number of bacteria decreased, but increased again at 1 h after oral care in the control group; however, in the intervention group, bacterial growth was inhibited up to 3 h after oral care. The number of bacteria in the oral cavity was significantly different between the intervention and control groups at 1, 2, and 3 h after oral care.

### Changes in the balance of the oral microbiota in the intervention group

**Fig. 4:**
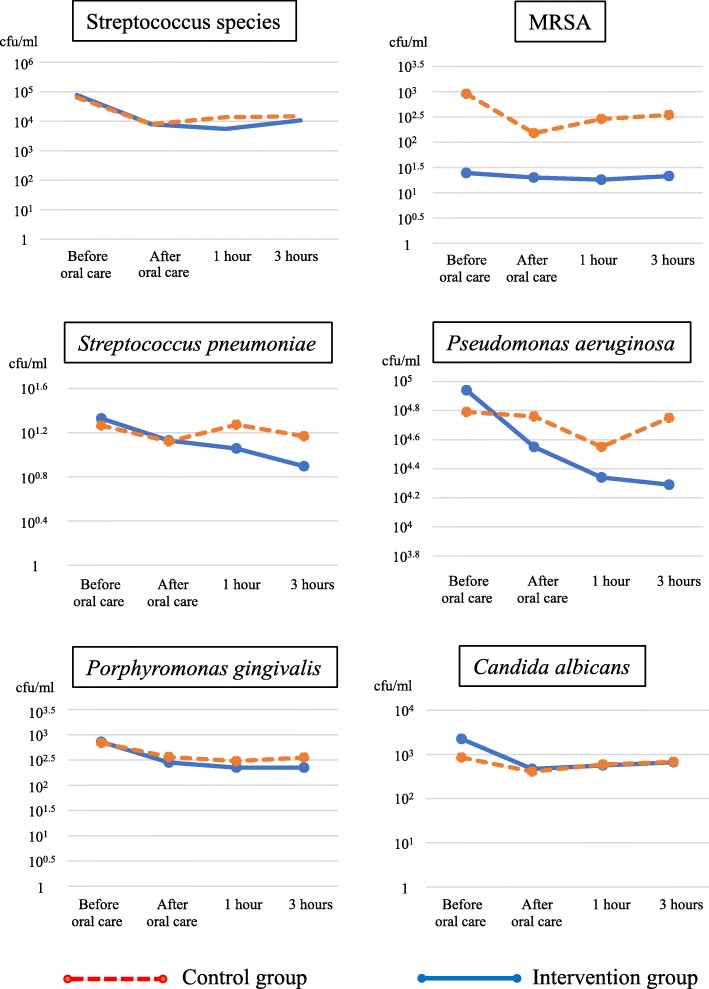
Changes in the count of each microorganism before and after oral care in the intervention and control groups. Each microorganism decreased at 1–3 h after application of povidone iodine, suggesting that this disinfectant does not disturb the oral microbiota or promote the growth of bacteria resistant to antibiotics (e.g., MRSA) or fungi

The number of streptococci, MRSA, *S. pneumoniae*, *P. aeruginosa*, *P. gingivalis*, and *C. albicans* was decreased at 1–3 h after oral care in the intervention group (Fig. 4). These findings suggest that povidone iodine inhibited the growth of all microorganisms tested and did not disturb the balance of the oral microbiota.

## Discussion

One of the main causes of VAP is thought to be aspiration of oropharyngeal fluid containing pathogenic microorganisms. Based on this premise, researchers have attempted to reduce VAP through oral care. Munro and Ruggiero [5], Pobo et al. [10], and Lorente et al. [11] conducted randomized controlled studies of the effect of tooth brushing on VAP prevention. They concluded that mechanical tooth brushing is not effective in preventing VAP. In another study, Mori et al. [12] reported that swabbing with povidone iodine gargle, tooth brushing, and irrigation with 300 mL of acidic water decreased a risk of VAP in 1252 mechanically ventilated patients compared with 414 patients who did not receive these procedures. Sone et al. [13] also described that tooth brushing, rinsing with tap water, and application of 0.12% chlorhexidine could decrease frequency of VAP. However, their studies were conducted with historical controls. Moreover, it remained unclear whether their oral care procedures actually reduced oral bacteria and how long the effects of oral care lasted. Hayashida et al. [14] reported that number of bacteria in the oropharyngeal fluid increased rapidly after intubation without growth of dental plaque, and stated that dental plaque was not a main reservoir of oropharyngeal bacteria in mechanically intubated patients. Funahara et al. [15] also described that the number of bacteria in the saliva increased after surgery but was not related to dental status such as amount of dental plaque and number of teeth.

Hayashida et al. [14] reported that irrigation with tap water reduces oral bacteria in ventilated patients, but the number of bacteria in oropharyngeal fluid increased within 3 h after irrigation. Funahara et al. described that topical tetracycline ointment on the tongue reduces the number of bacteria in oropharyngeal fluid for about 6 h [16]; furthermore, in a multicenter randomized clinical trial, topical application of tetracycline ointment on the tongue every 6 h for 24 h after surgery significantly prevented surgical site infection in patients undergoing oral cancer surgery with flap reconstruction and tracheotomy [17]. Some studies have investigated the effects of oral decontamination on VAP prevention. Rodriguez-Roldán et al. [18] reported that topical application of a paste containing tobramycin, amphotericin B, and polymyxin E in the oral cavity reduced the risk of VAP in 13 ventilated patients, although the overall mortality was not improved. Abele-Horn et al. [19] also reported that the incidence of VAP was reduced by topical administration of that same paste in 58 ventilated patients. Bergmans et al. [20] reported that topical antimicrobial prophylaxis consisting of an orabase paste containing gentamycin, colistin, and vancomycin reduced the risk of VAP in 92 patients. However, these studies failed to demonstrate the effect of topical antibiotics on decreasing mortality rate or hospital day. Furthermore, antibiotic administration may promote the emergence of resistant bacteria; therefore, the topical use of antibiotics is not recommended for mechanically ventilated patients.

One meta-analysis has shown that topical 0.12% chlorhexidine is effective in preventing VAP [21]. Although topical application of 0.12% chlorhexidine is a standard procedure in patients undergoing mechanical ventilation, its use in the oral mucosa is not approved in Japan because of reports of anaphylactic shock. Similar to chlorhexidine, povidone iodine has antibacterial activity and is approved for use in the oral cavity. However, it is not commonly used to prevent VAP. It has been suggested that povidone-iodine is cytotoxic to normal mucosal cells, and it has been pointed out that prolonged use of povidone iodine may cause tooth coloring. The CDC guidelines 2017 [22] recommend the use of iodine before wound closure during surgery, so we believe that the usefulness of the disinfecting effect outweighs the cytotoxicity concerns.

This randomized controlled study showed that topical application of 10% povidone iodine—an alternative to 0.12% chlorhexidine in Japan—after cleaning and irrigation of the oral cavity inhibited bacterial growth in the oropharyngeal fluid of patients on mechanical ventilation while not disrupting the balance of the oral microbiota. The present study showed that povidone iodine reduced the number of oral bacteria for at least 3 h after oral care in patients undergoing mechanical ventilation. Furthermore, the results of real-time PCR showed that topical povidone iodine did not disturb the balance of the oral microbiota or promote the growth of bacteria resistant to antibiotics (e.g., MRSA) or fungi. These findings suggest that topical application of povidone iodine is a simple and safe method to reduce oral bacteria for a longer time that could be used as standard prophylaxis against VAP in Japan—similar to 0.12% chlorhexidine globally. In this study, we investigated only up to 3 h after application, so it would be necessary to study for a longer time to establish an appropriate oral care method.

This study has some limitations. First, the sample size was small and the last measurements were obtained at only 3 h after oral care. Second, the outcome of the study was bacterial count, not the development of VAP. Therefore, we cannot conclude whether topical application of povidone iodine reduces the frequency of VAP, only that it inhibits bacterial growth in the oral cavity of patients undergoing mechanical ventilation. Further investigation is necessary to tackle these issues.

## Conclusions

Topical application of povidone iodine reduced the number of oral bacteria for at least 3 h after oral care in patients undergoing mechanical ventilation, and did not promote the growth of bacteria resistant to antibiotics or fungi. This is a simple and safe method to reduce oral bacteria for a longer time in Japan—similar to 0.12% chlorhexidine globally.

## Data Availability

The datasets used and analyzed during the current study are available from the corresponding author on reasonable request.

## Abbreviations

IHI: Institute for Healthcare Improvement
JSICM: Japanese Society of Intensive Care Medicine
MRSA: Methicillin-resistant *Staphylococcus aureus*
PCR: Polymerase chain reaction
UMIN-CTR: University Hospital Medical Information Network Clinical Trials Registry
VAP: Ventilator-associated pneumonia
